# Optimising Terpene Synthesis with Flow Biocatalysis

**DOI:** 10.1002/ejoc.201601388

**Published:** 2017-01-13

**Authors:** Xiaoping Tang, Rudolf K. Allemann, Thomas Wirth

**Affiliations:** ^1^ School of ChemistryCardiff UniversityPark Place, Main BuildingCF10 3ATCardiffUK

**Keywords:** Biocatalysis, Enzyme catalysis, Flow chemistry, Segmented flow, Terpenoids, Synthetic methods

## Abstract

Sesquiterpenes are an important family of natural products, many of which exhibit important pharmaceutical and agricultural properties. They are biosynthesised from farnesyl diphosphate in sesquiterpene synthase catalysed reactions. Here, we report the development of a highly efficient segmented flow system for the enzyme‐catalysed continuous flow production of sesquiterpenes. Design of experiment (DoE) methods were used to optimise the performance of the flow biocatalysis, and quantitative yields were achieved by using an operationally simple but highly effective segmented flow system.

## Introduction

**Scheme 1:**
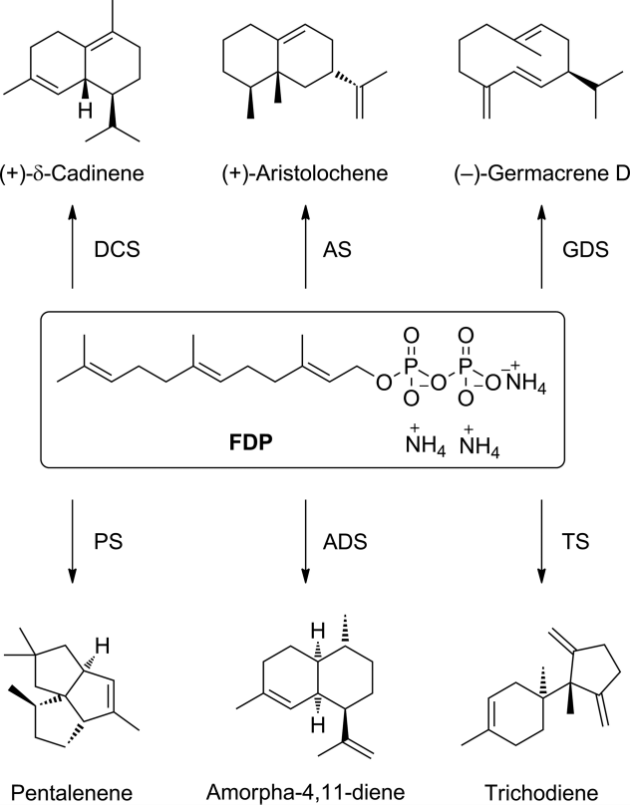
Examples of sesquiterpenes generated from FDP by terpene synthases.

Sesquiterpenes are one of the largest families of natural products. More than 300 distinct 15‐carbon skeletons and thousands of oxidised and other derivatives can be found in plants and microorganism with often interesting pharmaceutical and agricultural activities.^1^ An efficient synthetic route to these compounds is therefore highly desirable. Given the complex structure of sesquiterpenes, their chemical synthesis is often lengthy and low yielding.^2^ Despite their structural diversity, all sesquiterpenes are synthesised in nature from the common linear precursor farnesyldiphosphate (FDP) (**1**) by sesquiterpene synthases (Scheme 1).^3^ Sesquiterpene synthases generate the polycyclic core structures of sesquiterpenes, often with several stereogenic centres, in one step through a series of highly sophisticated and elegant reaction cascades that involve cyclisations, additions and rearrangements. The exquisite regio‐ and stereocontrol of sesquiterpene synthases make these enzymes attractive catalysts for the synthesis of this class of natural products.

The efficiency and synthetic utility of terpene synthases is somewhat limited by the slow release of the reaction products. The high hydrophobicity of sesquiterpenes limits their solubility in water^4^ and the aqueous incubation buffer quickly reaches saturation, causing the enzymatic reaction to stall. Pre‐steady‐state kinetic studies of trichodiene synthase showed that the enzymatic conversion of FDP to the sesquiterpene trichodiene is about 40 times faster than the rate of product release.^5^ One way to push the equilibrium towards product is the use of an organic solvent to continuously extract the product from the aqueous phase. Such two‐phase systems of immiscible liquids are typically used for sesquiterpene biosynthesis in conventional batch protocols. However, due to the limited contact surface area in a traditional tank system, the mass transfer rate between the two phases is low. This not only causes difficulties in scale up but also requires extended reaction times. Intense mixing by force could increase the mass transfer rate, but the resulting shear force and overexposure to the organic phase can lead to enzyme denaturation and loss of enzyme activity.^6^ With traditional stirring protocols, control of the mixing degree of the two liquids is limited.

## Results and Discussion

In a segmented flow system, the interfacial area between the two liquids is exquisitely controlled by the size of the solvent segments.^7^ Many researchers, including us, have utilised this to enhance mixing and accelerate reactions.^8^ The synthetic utility of terpene synthase catalysed reactions should be significantly improved in such systems, which allow maximal mass transfer rates without causing enzyme deactivation. The development of a continuous flow system also offers attractive scale up options for the production of high‐value sesquiterpenes.

**Figure 1:**
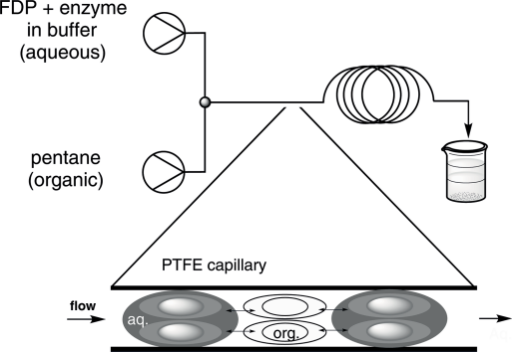
Segmented flow system for enzymatic terpene synthesis. The double‐ended arrow represents the diffusion between two liquids. The circles represent convective flow inside each solvent segment.

Based on earlier work,^9^ we designed a segmented flow system for terpene synthases (Figure 1), in which the two immiscible liquids enter the capillary tubing reactor through a T‐mixer and generate alternating liquid segments. This results in a much‐enhanced surface‐to‐liquid volume ratio and thereby generates a higher mass‐transfer rate; moreover, the shear forces between the capillary wall and the axis of segment causes intense internal circulation inside each segment. This convective motion renews the interfacial area, which augments the concentration gradient of the product and thereby facilitates the diffusive penetration through the interface.^10^ When exiting the reactor, the mixture is collected and the two immiscible liquids separate under gravity.

Pentane was chosen as the organic solvent for the segmented flow extraction. Sesquiterpenes have good solubility in pentane and the enzyme did not show any deactivation when exposed to this solvent. No product was observed when other solvents such as dichloromethane or ethyl acetate were used. Diethyl ether and toluene are also suitable for extraction of the enzymatic reaction; however, other chemicals from the incubation buffer such as 2‐mercaptoethanol were also found in the organic phase. With pentane, a clean extraction was achieved and only the sesquiterpene product was observed in the organic phase.

We chose *aristolochene synthase* (AS) as the model enzyme and premixed it with FDP under optimised incubation buffer conditions.^11^ Experiments without premixing showed no difference in product yields, which confirms that the reaction rate is largely dependent on the removal of product. The pH 7.5 incubation buffer contained Mg^2+^, which is essential for terpene synthase activation, and 2‐mercaptoethanol to prevent enzyme aggregation.

The reaction mixture was collected into a flask without additional separation steps. Pentane (Log *P* = 3.4) does not form an emulsion with the aqueous phase and can be separated quickly by gravitation. Our previous study showed that there was no difference in product yield whether the enzymatic reaction was quenched with ethylenediaminetetraacetic acid (EDTA) or not.^9^ We also investigated the effect of enzyme concentration and found that there was no significant reduction in yield when the concentration of AS was halved compared with the standard batch reaction conditions.^12^

A successful segment flow system for terpene synthesis requires an optimal segment distribution and, hence, after establishing the optimal reaction set‐up, the segment size was optimised. Segments that were too large resulted in inefficient mass transfer, whereas overly small segments resulted in excessive exposure to the organic solvent and deactivation of the enzyme. The three parameters known to influence segment distribution[10c] were optimised, namely: (i) the internal diameter (ID) of the capillary tubing reactor, (ii) the ratio between the two liquids, and (iii) the reaction time, which can also be interpreted as linear flow velocity.

**Figure 2:**
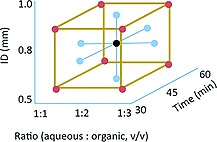
Face‐centred design for first set of experiments. Each point represents a different set of reaction conditions.

The traditional one variable at a time (OVAT) method often leads to the identification of a local optimum only so the reaction was optimised by using a design of experiment (DoE) method. The DoE method takes the interaction among different variables into account and is much more efficient than OVAT in terms of experimental effort.^13^ For the first set of experiments, we used a face‐centred design. Three levels for each parameter were set (Figure 2) and 15 reaction conditions were screened.

**Figure 3:**
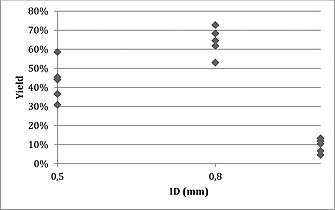
Yield depending on reactor internal diameter (ID).

All reactions were performed in random order to avoid systematic errors and each reaction parameter was plotted against product yield. From the results presented in Table 1 it is clear that the internal diameter of the reactor (ID) had the largest effect on product yield. There is a relatively narrow yield distribution with each tubing size, as illustrated in Figure 3. There was a clear decrease in product formation with the 1.0 mm ID reactor. Wider tubing on the other hand generated larger segments and an insufficient interfacial area. There were also signs of enzyme deactivation as a consequence of excessive exposure to pentane. The experimental conditions presented in entries 11 and 13 involved the same reactor (ID = 0.5 mm); in these cases, as the ratio of pentane increased the product yield decreased.

**Table 1:** Results of DoE experiments for AS in flow. Yields were analysed by GC and calculated by using α‐humulene as internal standard

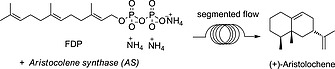				
Entry	Solvent ratio	Tubing ID	Reaction time	Yield
	(aqueous/organic, v/v)	[mm]	[min]	[%]
1	1:2	0.8	30	64
2	1:2	0.8	60	68
3	1:1	0.8	45	62
4	1:3	0.8	45	73
5	1:1	0.5	30	44
6	1:1	1	30	13
7	1:1	0.5	60	59
8	1:1	1	60	12
9	1:3	0.5	60	37
10	1:3	1	60	10
11	1:2	0.5	45	45
12	1:2	1	45	7
13	1:3	0.5	30	31
14	1:3	1	30	5
15	1:2	0.8	45	53

Based on the results of these first experiments, two data points (Table 1, entries 4 and 7) were selected for further experiments. The conditions described in entry 4 resulted in the highest yield of all experiments with an ID = 0.8 mm reactor, but lower ratio of pentane led to lower yields. Therefore, the reactor and ratio were kept but the time of the reaction was modified further (Table 2, entries 1–5) but no increase of yield was observed The ratio of aqueous to organic phase was extended to 1:4, but again the yield did not improve. The highest yield for tubing with ID = 0.5 mm is shown in Table 1, entry 7. For this tubing, segments were smaller than with ID = 0.8 mm, and further reducing the size of segment by increasing the ratio showed signs of enzyme deactivation; for the follow‐up experiment the reaction time was therefore extended. As the results show, time had a linear effect on yield; increasing the time to 90 min gave nearly quantitative yield (Table 2, entry 9), which is a marked improvement on all previous procedures.^8^

**Table 2:** Optimisation experiments for AS in flow. Yields were analysed by GC and calculated by using α‐humulene as internal standard

Entry	Solvent ratio	Tubing ID	Reaction time	Yield
	(aqueous/organic, v/v)	[mm]	[min]	[%]
1	1:3	0.8	30	67
2	1:3	0.8	60	58
3	1:4	0.8	45	56
4	1:4	0.8	57	65
5	1:4	0.8	80	57
6	1:1	0.5	68	72
7	1:1	0.5	75	80
8	1:1	0.5	83	82
9	1:1	0.5	90	96

**Scheme 2:**
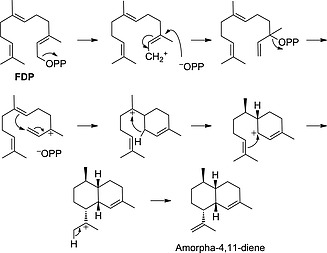
Mechanism for the conversion of FPP into amorpha‐4,11‐diene by *amorphadiene synthase* (ADS).

**Scheme 3:**
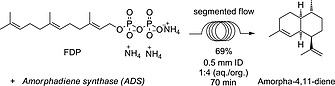
Amorpha‐4,11‐diene synthesis in flow.

After establishing the optimal conditions for AS, we used the same DoE principal for *amorphadiene synthase* (ADS), a sesquiterpene synthase from the plant A. *annua* that catalyses the conversion of FDP into amorpha‐4,11‐diene (Scheme 2), a key intermediate in the biosynthesis and the chemical synthesis of artemisinin.^14^ Artemisinin‐based combination therapy (ACT) is the first‐line treatment of malaria recommended by the World Health Organization (WHO).^15^ Unlike the fungal enzyme aristolochene synthase,^16^ ADS is less heat stable. The same segmented flow approach described above for AS, generated an almost 70 % yield of amorpha‐4,11‐diene using ADS at 2 µm concentration (Scheme 3).^17^ Amorpha‐4,11‐diene can be produced by fermentation,^18^ but the continuous‐flow system offers a highly attractive alternative for the production of this intermediate of artemisinin synthesis.

## Conclusions

The DoE method used for optimising the flow synthesis of terpenes led to the identification of optimal reaction conditions in a minimal number of experiments. Careful control of the physical interactions between the enzymes and the organic phase in segmented flow systems allows a high mass‐transfer rate without enzyme deactivation and leads to high product yields of high‐value terpenes.

## Experimental Section

**Transformation of *E. coli* BL21 with cDNA for Wild‐type AS:** *E. coli* BL21 competent cells (stored at –80 °C) were slowly defrosted in ice. Vector containing a cDNA for AS and resistance for ampicillin (1 µL) was added to the cells. After leaving on ice for 20 min, the mixture was submitted to thermal shock in a water bath at 40 °C for 35 s and returned to ice for 2 min. LB media (1 mL, sterilised) was added to the transformed cells under flame and the solution was shaken (150 rpm) at 37 °C for 1 h. The cells were separated from the media by centrifuging (6000 rpm) for 1 min. The cells were re‐suspended in a minimum amount of LB media and the mixture was spread on an ampicillin‐agar plate under flame. The plate was incubated at 37 °C for 12 h.

**Overexpression of AS:** To a solution of ampicillin (10 mg) in sterilised LB media (100 mL), a single colony from the plate was added. The media was incubated at 37 °C overnight. This overnight culture (10 mL) was added to sterilised LB media (500 mL) containing ampicillin (50 mg). The resulting mixture was incubated at 37 °C and the growth of bacteria was monitored by checking the OD of 1 mL of media at 600 nm, when reaching 0.6. The culture was induced by the addition of isopropyl‐1‐thio‐β‐d‐galactopyranoside (60 mg). The induced culture was incubated at 37 °C for 3 h. The solutions were centrifuged at 5000 rpm for 20 min, the supernatant was discarded, and the pellets were stored at –20 °C.

**Purification of AS:** The pellet was defrosted on ice and resuspended in cell lysis buffer (50 mL). The mixture was sonicated in an ice bath (3 min with 5 s on/10 s off cycles). The resulting mixture was centrifuged at 5000 rpm for 10 min, the supernatant was discarded, and the pellet was resuspended in fresh cell lysis buffer (70 mL). The solution was cooled in an ice bath and taken to pH 11.5 by adding NaOH (5 m). After stirring for 20 min at 4 °C, the pH of the mixture was carefully adjusted to 8 by adding HCl (1.0 m). The mixture was stirred for 30 min and centrifuged at 15000 rpm at 4 °C for 30 min. The supernatant was purified by anion Exchange Q‐SepharoseTM (Amersham Pharmacia BiotechTM) high‐performance (2.5 × 20 cm) column. The absorbance of the fractions was measured at 280 nm to identify fractions containing protein. The supernatant was loaded in the column and cell lysis buffer (150 mL) was then used to remove any unbound protein. Protein was eluted with an aqueous NaCl solution (500 mL gradient from 0.1 to 0.6 m) and then the column was washed with aqueous NaCl solution (200 mL, 1 m) to elute any remaining protein in the column. The presence of protein was confirmed by SDS‐PAGE electrophoresis. The fractions containing protein were combined and dialysed in dialysis buffer by using a Spectrum^TM^ Spectra/Por molecular porous dialysis membrane (MW = 3500 cutoff) at 4 °C for 24 h. The resulting protein solution was concentrated to 10 mL at 1 bar in Amicon^TM^ ultrafiltration apparatus with a millipore 44.5 mm ultrafiltration membrane. The concentration of AS was determined by using the Bradford assay.^19^

**Synthesis of FDP:** 20 To a stirred solution of farnesol (0.75 mL, 3.0 mmol) in anhydrous DMF (40 mL) at 0 °C, 2,4,6‐collidine (2.38 mL, 18 mmol) and methansulfonyl chloride (0.46 mL, 6.0 mmol) were added. After 15 min, lithium chloride (590 mg, 12 mmol) was added. After 3 h, the reaction was quenched with water (30 mL) and the mixture was extracted with hexane (3 × 40 mL). The combined organic phases were washed with sat. aq. CuSO_4_, water and sat. aq. NaHCO_3_ solution, dried with MgSO_4_ and concentrated under reduced pressure. The crude chloride was used in the next step without further purification.

To a stirred solution of the crude chloride (850 mg) in anhydrous acetonitrile (30 mL), tris(tetrabutylammonium)hydrogen pyrophosphate (5.4 g, 6 mmol) was added. The reaction was stirred at room temperature for 16 h. Acetonitrile was removed under reduced pressure and the remaining yellow oil was dissolved in buffer (15 mL, 25 mm of NH_4_HCO_3_, 2 % 2‐propanol) and passed thought an ion exchange column DOWEX 40 W (NH_4_^+^ form). The eluent from the ion exchange column was monitored by TLC (2‐propanol/buffer/NH_4_OH = 6:2:2). Fractions containing product were collected and freeze dried. The yellow solid was diluted in buffer (15 mL) and the crude material was purified by reverse‐phase prep‐HPLC (150 × 21.2 mm Phenomenex Luna column, eluting with 10 % B for 20 min, then a linear gradient to 60 % B over 25 min and finally a linear gradient to 100 % B over 5 min; solvent A: 25 mm NH_4_HCO_3_ in water, solvent B: CH_3_CN, flow rate 5.0 mL/min, detecting at 220 nm). The fractions from prep‐HPLC were freeze dried and pure FDP (130 mg, 30 % yield) was obtained as a colourless solid.^21^ m.p. 156–160 °C (decomposition at 158 °C). ^1^H NMR (300 MHz, D_2_O): *δ* = 5.38 (t, *J* = 7.0 Hz, 1 H, C=CH), 5.16–5.00 (m, 2 H, 2 × C=CH), 4.39 (t, *J* = 6.5 Hz, 2 H, CH_2_OPP), 2.15–1.83 (m, 8 H, 4 × CH_2_), 1.65 (s, 3 H, CH_3_), 1.59 (s, 3 H, CH_3_), 1.54 (s, 3 H, CH_3_), 1.52 (s, 3 H, CH_3_) ppm. ^13^C NMR (75 MHz, D_2_O): *δ* = 154.3, 142.7, 136.5, 133.4, 124.3, 124.1, 119.7 (d, *J*_c,p_ = 7.5 Hz), 62.4, 38.6, 25.6, 25.5, 24.7, 16.8, 15.4, 15.1 ppm. ^31^P NMR (32 MHz, D_2_O): *δ* = –6.90 (d, *J*_P,P_ = 15.0 Hz), –10.40 (d, *J*_P,P_ = 15.0 Hz) ppm.

**Flow Synthesis:** The flow reactor was constructed from PTFE tubing (Diba, Kinesis Ltd) to a total volume of 2 mL. The two liquid streams, aqueous and organic, were introduced to the reactor through a T‐piece by using two syringe pumps (Fusion 100 Touch infusion syringe pump, KR Analytical Ltd), and the reaction mixture was collected in a glass beaker at exit. For each reaction, a combined total volume of 6 mL (three reactor volumes) for aqueous and organic solutions were made, the exact volume of each solution depends on the ratio of the reaction. During the reaction, only the third reactor volume was collected and analysed to ensure the reaction had reached steady state. For the reaction in 0.5 mm ID tubing, 1:1 ratio, 90 min: The first syringe (plastic) was loaded with 3 mL AS incubation buffer (20 mm Trizma, 5 mm 2‐mercaptoethanol, 15 % v/v glycerol, 3 mm MgCl_2_) containing 6 µm AS and 0.35 mm FDP and injected at a flow rate of 0.011 mL/min; the second syringe (glass) was loaded with 3 mL pentane containing 35 µm α‐humulene (internal standard) and injected at a flow rate of 0.011 mL/min. After 180 min, the reaction mixture was collected for 90 min and the organic layer was analysed by GC. The yield was calculated by comparing peak areas of product and internal standard.

## Supporting information

Supporting Information
