# Supplementary material for: Optimising Terpene Synthesis with Flow Biocatalysis
Source: European J Org Chem. 2017 Jan 13;2017(2):414–8. doi: 10.1002/ejoc.201601388 (PMC5324637; doi:10.1002/ejoc.201601388)
Supplement: Supplementary file 1 — Supporting Information [file EJOC-2017-414-s001.pdf]

*Eur. J. Org. Chem.* **2017** · ISSN 1099–0690

## *SUPPORTING INFORMATION*

***DOI:*** 10.1002/ejoc.201601388

***Title:*** Optimising Terpene Synthesis with Flow Biocatalysis

***Author(s):*** Xiaoping Tang, Rudolf K. Allemann,* Thomas Wirth*

# Optimising Terpene Synthesis with Flow Biocatalysis

Xiaoping Tang, Rudolf K. Allemann* and Thomas Wirth*


1. General Remarks S2

2. Synthesis of FDP S3

3. Enzyme preparation S5

4. Optimisation of AS in flow S7

5. Optimisation of ADS in flow S13

## 1. General Remarks

All chemicals were purchased from Sigma-Aldrich, Acros Chemicals or Alpha Aesar unless otherwise stated. Anhydrous acetonitrile was obtained from a MBraun SPS800 solvent purification system. Reactions were performed under $N_2$ atmosphere unless otherwise stated. Glassware was cleaned and dried at 200 °C for at least for 16 h before use.

$^{1}$H-NMR and $^{13}$C-NMR spectra were measured on a Bruker Avance 500 NMR spectrometer, Bruker Avance DPX400 spectrometer. $^{1}$H-NMR spectra are reported as chemical shifts in parts per million downfield from tetramethylsilane, multiplicity (s = singlet, d = doublet, t = triplet, q = quartet, m = multiplet, bs = broad singlet, bd = broad doublet), coupling constant, integration and assignment, respectively. All the coupling constants are reported in Hz. $^{13}$C NMR spectra were reported as chemical shift downfield from tetramethylsilane. $^{31}$P NMR spectra were recorded on Bruker Avance DPX400 spectrometer or Bruker Avance 500 NMR spectrometer and are reported in chemical shift downfield from 85% $H_3PO_4$ followed by multiplicity and coupling constant in Hz if appropriate. $EI^+$ mass spectra were measured on a Micromass LCT premiere XE mass spectrometer. $ES^-$ mass spectra were measured on a Micromass LCT premiere XE spectrometer fitted with a Waters 1525 Micro binary HPLC pump. Reverse phase HPLC was performed on a system comprising of a Dionex P680 pump and a Dionex UVD170U detector unit. GCMS was performed on a Hewlett Packard 6890 GC fitted with a J&W scientific DB-5MS column (30 m x 0.25 mm internal diameter) and a Micromass GCT Premiere detecting in the range m/z 50-800 in EI+ mode with scanning once a second with a scan time of 0.9 s.

Ion exchange resin DOWEX 40-W was received from Aldrich in H+ form. The resin was converted into ammonium form by washing with concentrated $NH_4OH$, then deionised water until the pH drops to 7, finally equilibrated with ion-exchange buffer (25 mM $NH_4HCO_3$ containing 2% i-PrOH).

## 2. Synthesis of FDP[1]

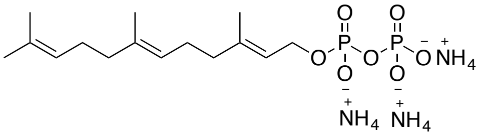

To a stirred solution of farnesol (0.75 mL, 3.0 mmol) in anhydrous DMF (40 mL) at 0 °C, 2,4,6-collidine (2.38 mL, 18 mmol) and methansulfonyl chloride (0.46 mL, 6.0 mmol) were added. After 15 min, lithium chloride (590 mg, 12 mmol) was added. After 3 h, the reaction was quenched by water (30 mL). The mixture was extracted with hexane (3 x 40 mL). The combined organic phases were washed with $CuSO_4$ (saturated aq.), water and saturated aq. $NaHCO_3$ solution, dried over $MgSO_4$ and concentrated under reduced pressure. The crude chloride was used in the next step without further purification.

To a stirred solution of the crude chloride (850 mg) in dry acetonitrile (30 mL), tris-(tetrabutylammonium)hydrogen pyrophosphate (5.4 g, 6 mmol) was added. The reaction was stirred at room temperature for 16 h. Acetonitrile was removed under reduced pressure, the remaining yellow oil was dissolved in buffer (15 mL, 25 mM of $NH_4HCO_3$, 2% isopropanol) and passed thought an ion exchange column DOWEX 40 W ($NH_4^+$ form). The eluent from the ion exchange column was monitored by TLC (isopropanol / buffer / $NH_4OH$ 6:2:2). Fractions containing product were collected and freeze dried. The yellow solid was diluted in buffer (15 mL). The crude was purified by reverse-phase prep-HPLC (150 × 21.2 mm Phenomenex Luna column, eluting with 10% B for 20 min, then a linear gradient to 60% B over 25 min and finally a linear gradient to 100% B over 5 min.; solvent A: 25 mM $NH_4HCO_3$ in water, solvent B: $CH_3CN$, flow rate 5.0 mL/min, detecting at 220 nm). The fractions from prep-HPLC were freeze dried and pure FDP was obtained as a colourless solid (130 mg, 30% yield).[2]

$^1$H NMR (300 MHz, $D_2O$): δ = 5.38 (t, $J$ = 7.0, 1 H, C=CH), 5.16 – 5.00 (m, 2 H, 2 x C=CH), 4.39 (t, $J$ = 6.5, 2 H, $CH_2$OPP), 2.15 – 1.83 (m, 8 H, 4 x $CH_2$), 1.65 (s, 3 H, $CH_3$), 1.59 (s, 3 H, $CH_3$), 1.54 (s, 3 H, $CH_3$), 1.52 (s, 3 H, $CH_3$) ppm.

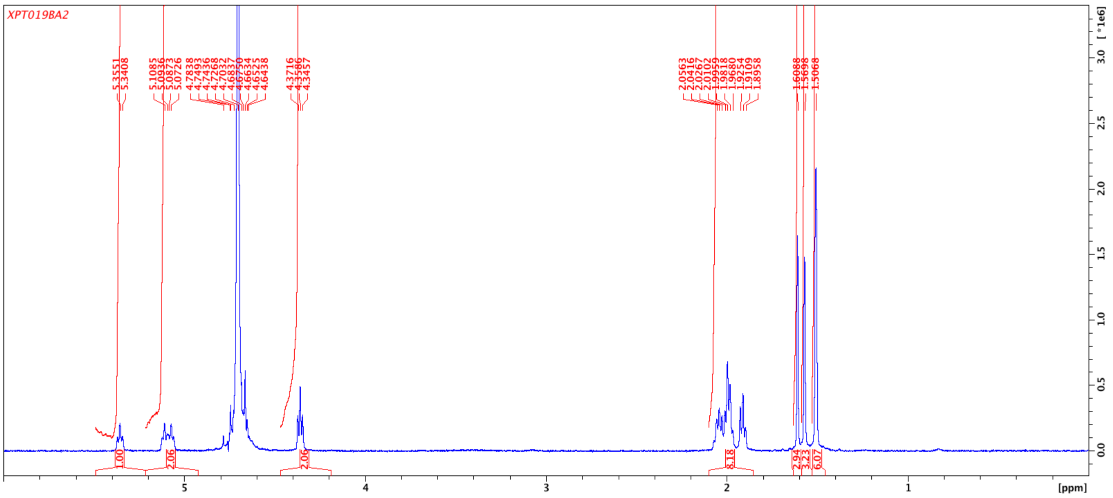

$^{31}P$ NMR (32 MHz, $D_2O$): -6.90 (d, $J_{PP}$ = 15.0), -10.40 (d, $J_{PP}$ = 15.0) ppm.

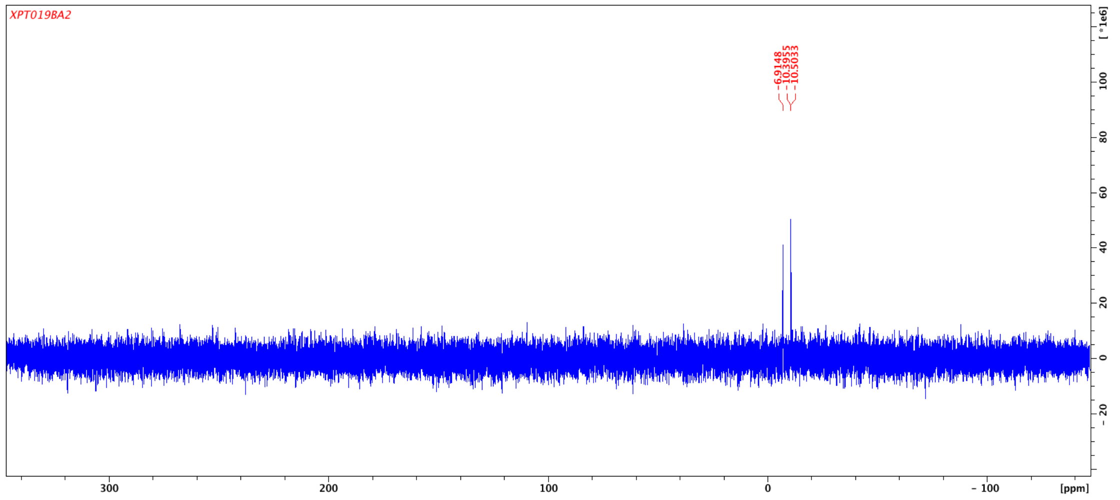

$^{13}C$ NMR (75 MHz, $D_2O$): δ = 154.3, 142.7, 136.5, 133.4, 124.3, 124.1, 119.7 (d, $J_{cp}$ = 7.5 Hz), 62.4, 38.6, 25.6, 25.5, 24.7, 16.8, 15.4, 15.1 ppm.

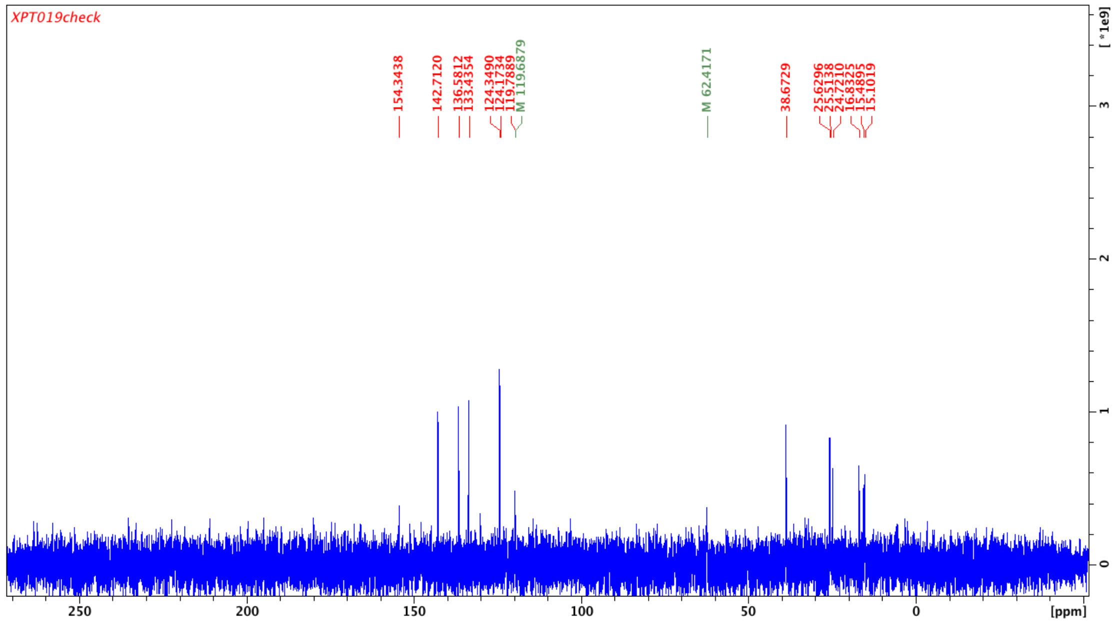

**m.p.**: 156-160˚C (decomposition observed at 158˚C)

# 3. Enzyme Preparation

## General methods

LB media was prepared by dissolving tryptone (10 g), yeast extract (5 g) purchased from Fluka and NaCl (10 g) in 1 L of deionised water.

Cell lysis buffer for AS was prepared by dissolving EDTA (5 mM), 2-mercaptoethanol (5 mM) and trizma-base (20 mM) in deionised water. The final pH was adjusted to 8.

Cell lysis buffer for ADS was prepared by dissolving trizma-HCl (50 mM), NaCl (500 mM), 2-mercaptoethanol (20 mM) and glycerol (10% v//v) in deionised water. The final pH was adjusted to 8.

Dialysis buffer for AS was prepared by dissolving trizma-base (20 mM) and 2-mercaptoethanol (5 mM) in deionised water. The final pH was adjusted to 8.

Dialysis buffer for ADS was prepared by dissolving HEPES (25 mM), NaCl (100 mM) and dithiothretol (1 mM) in deionised water. The final pH was adjusted to 7.5.

## 3.1 Preparation of AS[3]

### *3.1.1 Transformation of E. coli BL21 with cDNA for wild-type AS*

E. Coli BL21 competent cells (stored at –80 ˚C) was slowly defrosted in ice. Vector containing a cDNA for AS and resistance for ampicilline (1 µL) was added to the cells. After leaving on ice for 20 min, the mixture was thermally shock in a water bath at 40 ˚C for 35s and returned to ice for 2 min. LB media (1 mL, sterilised) was added to the transformed cells under flame and the solution was shaken (150 rpm) at 37 ˚C for 1 h. The cells were separated from the media by centrifuging the mixture (6000 rpm) for 1 min. The cells were re-suspended in the minimum amount of LB media and the mixture was spread in an ampicilline-agar plate under flame. The plate was incubated at 37 ˚C for 12 h.

### *3.1.2 Overexpression of AS*

To a solution of ampicilline (10 mg) in sterilised LB media (100 mL), a single colony from the plate was added. The media was incubated at 37 ˚C overnight. This overnight culture (10 mL) was added to sterilized LB media (500 mL) containing ampicilline (50 mg). The resulting mixture was incubated at 37 ˚C and the growth of bacteria was monitored by checking the OD of 1 mL of media at 600 nm, when reaching 0.6. The culture was induced by isopropyl-1-thio-β-D-galactopyranoside (60 mg). The induced culture was incubated at 37 ˚C for 3 h. The solutions were centrifuged at 5000 rpm for 20 min, the supernatant was discarded and the pellets were stored at –20 ˚C.

### *3.1.3 Purification of AS*

The pellet was de-frosted on ice and re-suspended in cell lysis buffer (50 mL). The mixture was sonicated in an ice bath (3 min with 5 s on/10 s off cycles). The resulting mixture was centrifuged at 5000 rpm for 10 min. The supernatant was discarded and the pellet was re-suspended in fresh cell lysis buffer (70 mL). The solution was cooled in ice bath and taken to pH = 11.5 by adding NaOH (5 M). After stirring for 20 min at 4 ˚C, the pH of the mixture was carefully adjusted to 8 by adding HCl (1.0 M). The mixture was stirred for 30 min and centrifuged at 15000 rpm at 4 ˚C for 30 min.

The supernatant was purified by anaion Exchange Q-SepharoseTM (Amersham Pharmacia BiotechTM) High Performance (2.5 × 20 cm) column. The absorbance of the fractions was measured at 280 nm to identify fractions containing protein. The supernatant was loaded in the column and then cell lyssis buffer (150 mL) was used to remove any unbound protein. Protein was eluted with an aqueous NaCl solution (500 mL gradient from 0.1 to 0.6 M) and then the column was washed with aqueous NaCl solution (200 mL, 1 M) to elute any remaining protein in the column. The presence of protein was confirmed by SDS-PAGE electrophoresis. Combine all the fractions with protein and dialyzed in dialysis buffer using Spectrum™ Spectra/Por molecular porous dialysis membrane (MW = 3500 cut off) at 4 ˚C for 24 h. The resulting protein solution was concentrated to 10 mL at 1 bar in Amicon™ ultrafiltration apparatus with a millipore 44.5 mm ultrafiltration membrane. The concentration of AS was determined by Bradford Assay.[4]

## 3.2 Preparation of ADS

### *3.2.1 Transformation of E. coli BL21 with cDNA for wild-type ADS*

Same procedure as AS.

### *3.2.2 Over expression of ADS*

The overnight culture was prepared the same way as AS. The resulting mixture was incubated at 37 ˚C and the growth of bacteria was monitored by checking the OD of 1 mL of media at 600 nm, when reaching 0.5. The culture was induced by isopropyl-1-thio-β-D-galactopyranoside (60 mg). The induced culture was incubated at 20 ˚C for 6 h. The solutions were centrifuged at 5000 rpm for 20 min, the supernatant was discarded and the pellets were stored at –20 ˚C.

### *3.2.3 Purification of ADS*

All the buffers for ADS purification were cooled on ice before use. Defrost the pellet on ice and re-suspended in cell lysis buffer (40 mL). After adding lysozyme (20 mg), the mixture was stirred at 4 ˚C for 30 min. The solution was sonicated in ice bath (3 min with 5 s on/10 s off cycles) then centrifuged at 5000 rpm for 10 min. The supernatant was loaded onto a $Ni^{2+}$ NTA column and eluted with a gradient of imidazole in cell lysis buffer (5 mM to 300 mM). The protein eluted at 100 mM imidazole. The presence of protein was confirmed by SDS-PAGE electrophoresis. Combine all the fractions with protein and dialyzed in dialysis buffer at 4 ˚C for 24 h. The resulting protein solution was concentrated to 10 mL final volume. The concentration of ADS was determined by Bradford Assay.

## 4. Optimisation of AS in flow

Incubation buffer for AS: Trizma-base (20 mM), 2-mercaptoethanol (5 mM), glycerol (15%, v/v), $MgCl_2$ (3 mM), pH = 7.5.

For all experiments, the aqueous solution was prepared by dissolving FDP (0.35 mM) and AS (6 $\mu$M) in the incubation buffer. The organic solvent is pentane with α-humulene (35 $\mu$M) as internal standard (IS). The reaction mixture was collected after reaching steady state (third reactor volume), the organic layer was directly analysed by GC.

Yield [%] = $A_p/A_{IS}$ * 10 * ratio

$A_p$: Area of product; $A_{IS}$: Area of internal standard; ratio: ratio of pentane to aqueous used for the reaction.

*Table S1: Results for the first set DoE*

| Entry | Ratio (Pentane ; aq., v/v) | Area Aristolochene | Area IS | Yield |
|---|---|---|---|---|
| 1 | 2 | 287 | 89 | 64% |
| 2 | 2 | 338 | 99 | 68% |
| 3 | 1 | 562 | 91 | 62% |
| 4 | 3 | 235 | 97 | 73% |
| 5 | 1 | 603 | 103 | 59% |
| 6 | 1 | 138 | 104 | 13% |
| 7 | 1 | 503 | 114 | 44% |
| 8 | 1 | 131 | 110 | 12% |
| 9 | 3 | 134 | 110 | 37% |
| 10 | 3 | 38 | 110 | 10% |
| 11 | 2 | 211 | 93 | 45% |
| 12 | 2 | 40 | 120 | 7% |
| 13 | 3 | 106 | 103 | 31% |
| 14 | 3 | 16 | 105 | 5% |
| 15 | 2 | 282 | 106 | 53% |

Selected GC chromatography:

*Table S1, entry 4:*

```
=====================================================================
Acq. Operator   : XPT
Acq. Instrument : Instrument 1                Location :   -
Injection Date  : 10/21/2015 11:40:06 AM
                                            Inj Volume : Manually
Acq. Method     : C:\CHEM32\1\METHODS\XPT_ISO155_SPLIT_20.M
Last changed    : 8/13/2015 2:40:06 PM by Xiaoping Tang
Analysis Method : C:\CHEM32\1\DATA\XPT\XPT038.D\DA.M (XPT_ISO155_SPLIT_20.M)
Last changed    : 10/21/2015 2:12:36 PM by XPT
                  (modified after loading)
Method Info     : Isothermal 155
                  Inlet temp 220
                  Split ratio 20
                  Oven: 155 degree C, 12 min
                  Detector temp 300
```

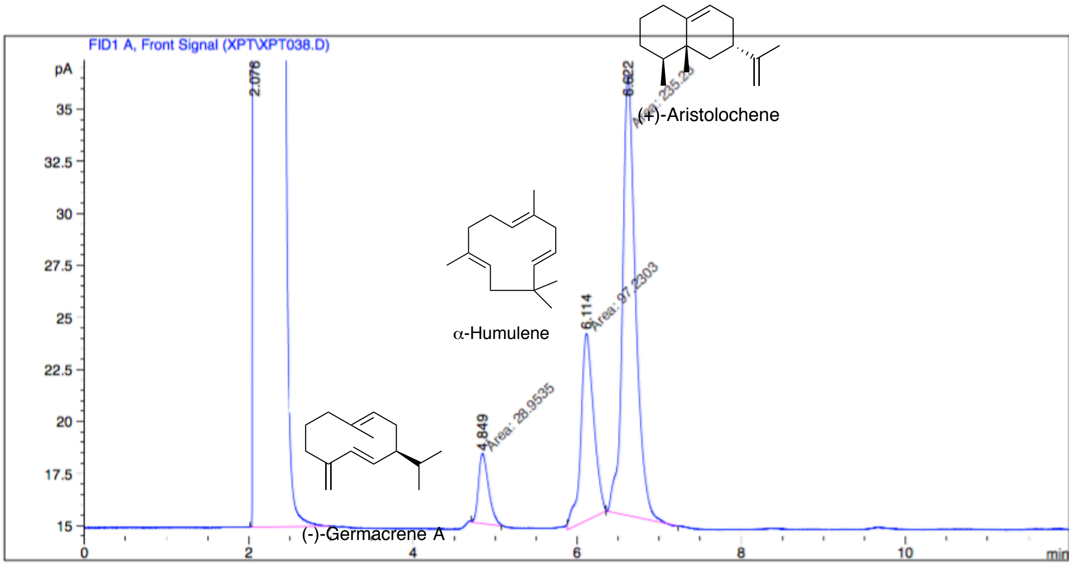


```
=====================================================================
                        Area Percent Report
=====================================================================

Sorted By              :      Signal
Multiplier             :      1.0000
Dilution               :      1.0000
Use Multiplier & Dilution Factor with ISTDs


Signal 1: FID1 A, Front Signal

Peak RetTime Type  Width     Area      Height     Area
  #   [min]        [min]    [pA*s]      [pA]        %
----|-------|----|-------|----------|----------|--------|
   1   2.076 BB S  0.1035 4.75621e6  7.16150e5  99.99240
   2   4.849 MM    0.1433   28.95352    3.36749   0.00061
   3   6.114 MM    0.1801   97.23034    8.99710   0.00204
```





```
Peak RetTime Type  Width     Area      Height     Area
  #   [min]        [min]    [pA*s]      [pA]        %
----|-------|----|-------|----------|----------|--------|
   4   6.622 MM    0.1853  235.22952   21.15911   0.00495

Totals :                  4.75657e6  7.16183e5


=====================================================================
                         *** End of Report ***
```

*Table S1, entry 5:*

```
=====================================================================
Acq. Operator   : XPT
Acq. Instrument : Instrument 1                    Location :   -
Injection Date  : 10/21/2015 3:00:40 PM
                                               Inj Volume : Manually
Acq. Method     : C:\CHEM32\1\METHODS\XPT_ISO155_SPLIT_20.M
Last changed    : 10/21/2015 11:52:10 AM by XPT
Analysis Method : C:\CHEM32\1\DATA\XPT\XPT039.D\DA.M (XPT_ISO155_SPLIT_20.M)
Last changed    : 10/21/2015 3:23:26 PM by XPT
                  (modified after loading)
Method Info     : Isothermal 155
                  Inlet temp 220
                  Split ratio 20
                  Oven: 155 degree C, 12 min
                  Detector temp 300
```

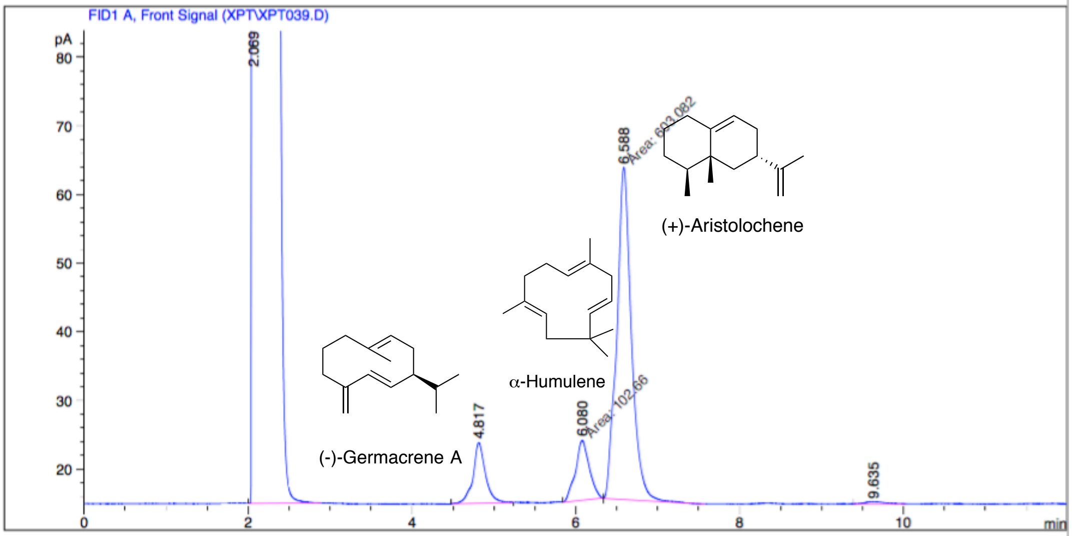


```
=====================================================================
                         Area Percent Report
=====================================================================

Sorted By             :      Signal
Multiplier            :      1.0000
Dilution              :      1.0000
Use Multiplier & Dilution Factor with ISTDs


Signal 1: FID1 A, Front Signal

Peak RetTime Type  Width     Area      Height     Area
  #   [min]        [min]    [pA*s]      [pA]        %
----|-------|----|-------|----------|----------|--------|
   1   2.069 BB S  0.0982 4.50692e6  7.08330e5 99.98216
   2   4.817 BB    0.1534   93.27118    8.82100  0.00207
   3   6.080 MM    0.1958  102.65993    8.73888  0.00228
```

Instrument 1 10/21/2015 3:24:16 PM XPT Page 1 of 2

Data File C:\CHEM32\1\DATA\XPT\XPT039.D
Sample Name: XPT039

```
Peak RetTime Type  Width     Area      Height     Area
  #   [min]        [min]    [pA*s]      [pA]        %
----|-------|----|-------|----------|----------|--------|
   4   6.588 MM    0.2075  603.08234   48.43327  0.01338
   5   9.635 BB    0.2325    5.06349 3.20199e-1  0.00011

Totals :                  4.50772e6  7.08396e5


=====================================================================
                          *** End of Report ***
```

*Table S2: Results for follow-up experiments*

| Entry | ID (mm) | Ratio (Pentane:aq. v/v) | Time (min) | Area Aristolochene | Area IS | Yield |
|---|---|---|---|---|---|---|
| 1 | 0.8 | 3 | 45 | 235 | 97 | 73%[a] |
| 2 | 0.8 | 3 | 45 | 5617669 | 2503313 | 67% |
| 3 | 0.8 | 2 | 30 | 48402 | 15053 | 64% |
| 4 | 0.8 | 2 | 30 | 287 | 89 | 64%[a] |
| 5 | 0.8 | 3 | 30 | 4009360 | 1787672 | 67% |
| 6 | 0.8 | 3 | 60 | 4547556 | 2358681 | 58% |
| 7 | 0.8 | 4 | 45 | 2364796 | 1693432 | 56% |
| 8 | 0.8 | 4 | 57 | 2233877 | 1374439 | 65% |
| 9 | 0.8 | 4 | 80 | 2018980 | 1423123 | 57% |
| 10 | 0.5 | 1 | 60 | 82590 | 11394 | 72% |
| 11 | 0.5 | 1 | 75 | 14705501 | 1830612 | 80% |
| 12 | 0.5 | 1 | 83 | 12586232 | 1526890 | 82% |
| 13 | 0.5 | 1 | 90 | 16280193 | 1698462 | 96% |

a: Analysed by GC-FID

We started the follow-up experiment by repeating a few experiments to check the reproducibility (entries 1 vs. 2; entries 3 vs. 4). All yields are in good agreement. This set of experiments were analysed by GC-MS, the yield was compared with GC-FID and the error range is within 5%.

Selected GC chromatography:

*Table S2, entry 4:*

Acq. Operator : XPT
Acq. Instrument : Instrument 1 Location : -
Injection Date : 10/20/2015 12:03:00 PM
Inj Volume : Manually
Acq. Method : C:\CHEM32\1\METHODS\XPT_ISO155_SPLIT_20.M
Last changed : 10/20/2015 11:33:34 AM by XPT
(modified after loading)
Analysis Method : C:\CHEM32\1\DATA\XPT\XPT035.D\DA.M (XPT_ISO155_SPLIT_20.M)
Last changed : 10/20/2015 1:32:42 PM by XPT
(modified after loading)
Method Info : Isothermal 155
Inlet temp 220
Split ratio 20
Oven: 155 degree C, 12 min
Detector temp 300

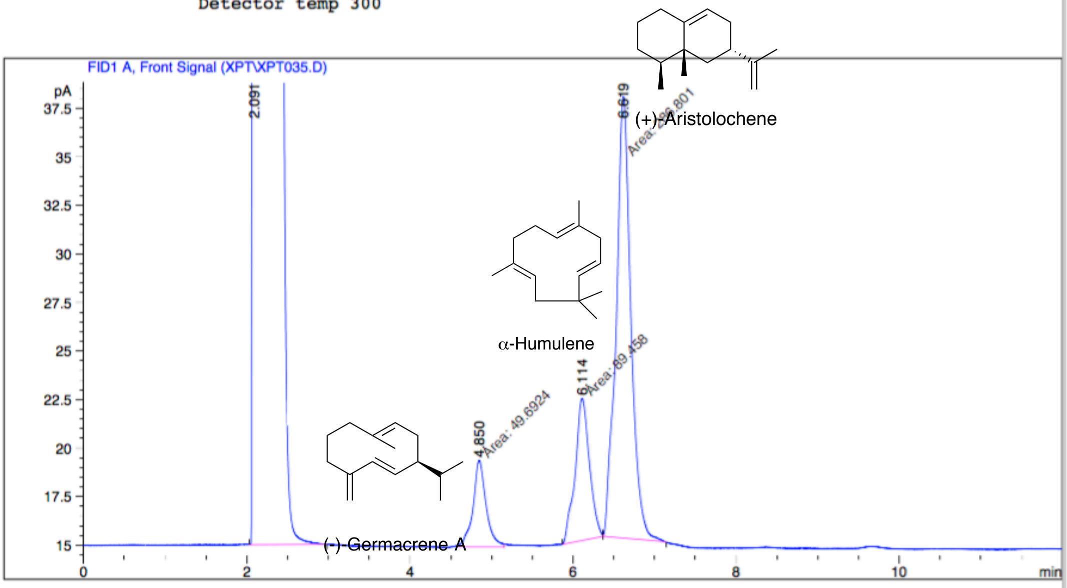


Area Percent Report

Sorted By : Signal
Multiplier : 1.0000
Dilution : 1.0000
Use Multiplier & Dilution Factor with ISTDs

Signal 1: FID1 A, Front Signal

| Peak # | RetTime [min] | Type | Width [min] | Area [pA*s] | Height [pA] | Area % |
|---|---|---|---|---|---|---|
| 1 | 2.091 | BB S | 0.0976 | 4.61242e6 | 7.31372e5 | 99.99077 |
| 2 | 4.850 | MM | 0.1857 | 49.69238 | 4.45895 | 0.00108 |





| Peak # | RetTime [min] | Type | Width [min] | Area [pA*s] | Height [pA] | Area % |
|---|---|---|---|---|---|---|
| 3 | 6.114 | MM | 0.2040 | 89.45798 | 7.31004 | 0.00194 |
| 4 | 6.619 | MM | 0.2104 | 286.80148 | 22.72162 | 0.00622 |

Totals : 4.61285e6 7.31406e5

*** End of Report ***

*Table S2, entry 5:*

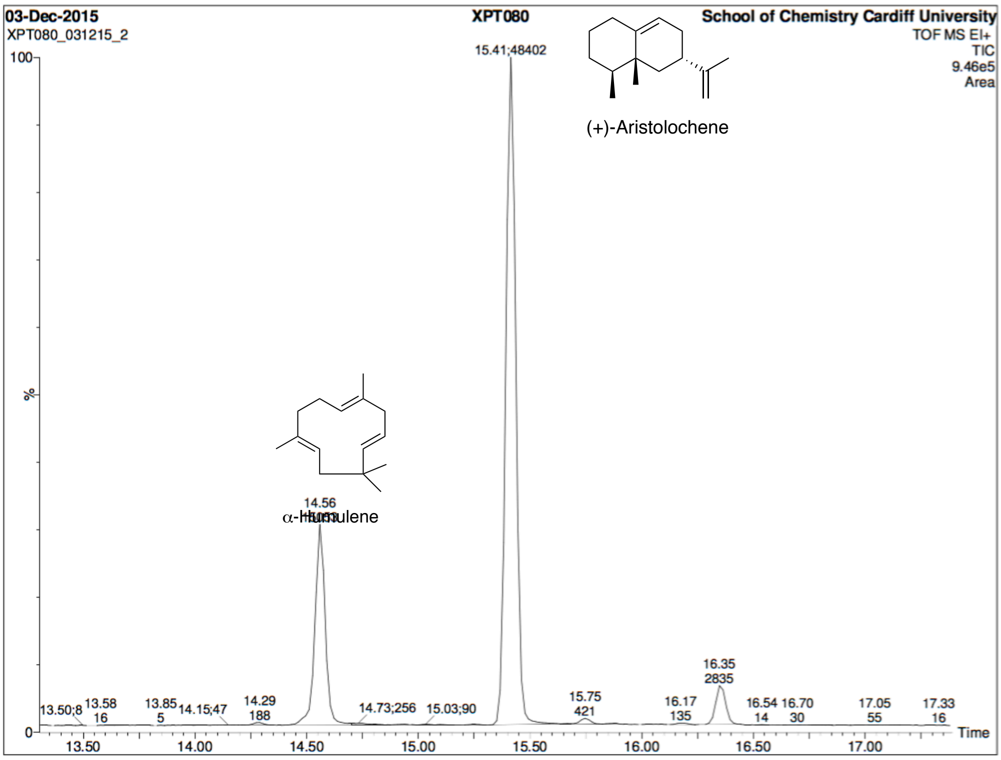


*Table S2, entry 13:*

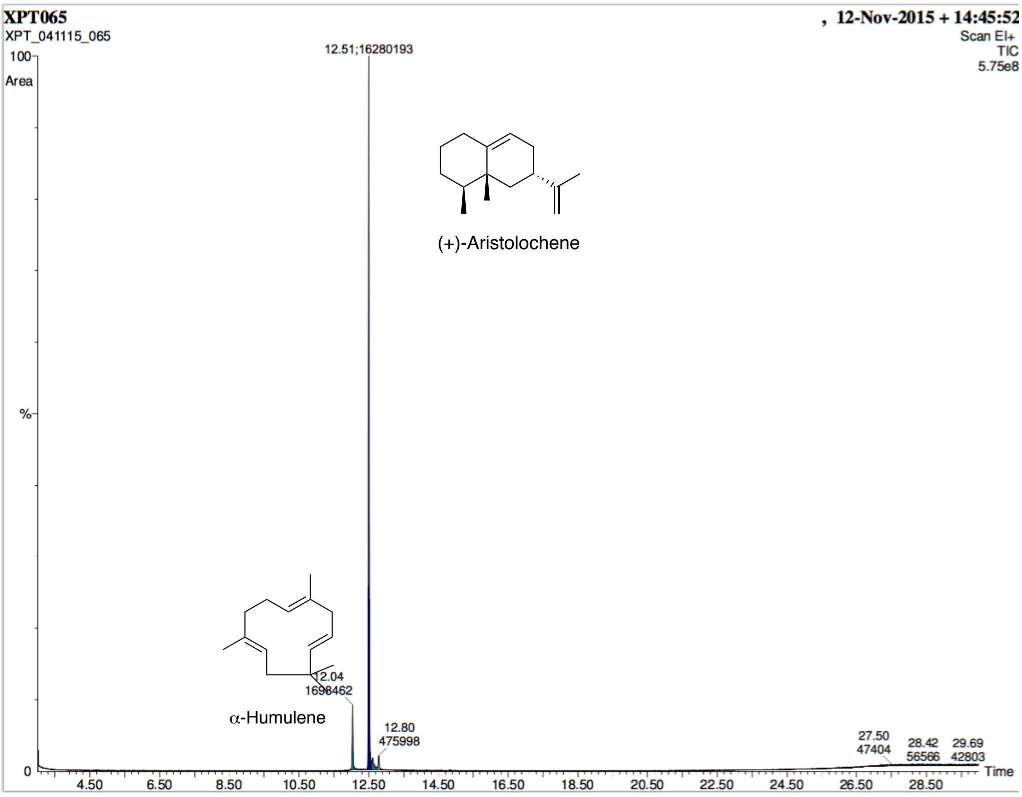

## 5. Optimisation of ADS in flow

Incubation buffer for ADS: HEPES (25 mM), dithiothretol (1 mM), $MgCl_2$ (5 mM), pH = 7.5.

For all the experiments, the aqueous solution was made by dissolving FDP (0.35 mM) and ADS (2 $\mu$M) in the incubation buffer. The organic solution is pentane with α-humulene (35 $\mu$M) as internal standard (IS). The reaction mixture was collected after reaching steady state (third reactor volume), organic layer was directly analysed by GC.

Yield [%] = $A_p/A_{IS}$ * 10 * ratio

$A_p$: Area of product; $A_{IS}$: Area of internal standard; ratio: ratio of pentane to aqueous used for the reaction.

*Table S3: Results for ADS optimisation*

| | Ratio (Pentane:aq. v/v) | ID (mm) | Time (min) | Area Amorphadiene | Area IS | Yield |
|---|---|---|---|---|---|---|
| 1 | 2 | 0.5 | 30 | 139 | 91 | 31% |
| 2 | 2 | 0.8 | 30 | 58 | 70 | 17% |
| 3 | 2 | 0.5 | 60 | 253 | 88 | 58% |
| 4 | 2 | 0.8 | 60 | 83 | 73 | 23% |
| 5 | 4 | 0.5 | 30 | 28 | 70 | 16% |
| 6 | 4 | 0.8 | 30 | 14 | 65 | 9% |
| 7 | 4 | 0.5 | 60 | 127 | 80 | 64% |
| 8 | 4 | 0.8 | 60 | 43 | 73 | 24% |
| 9 | 3 | 0.5 | 45 | 59 | 70 | 25% |
| 10 | 3 | 0.8 | 45 | 68 | 65 | 31% |
| 11 | 4 | 0.5 | 70 | 148 | 86 | 69% |

Selected GC chromatography

*Table S3, entry 3:*

```
=====================================================================
Acq. Operator   : Xiaoping
Acq. Instrument : Instrument 1                Location :   -
Injection Date  : 1/19/2016 11:15:29 AM
                                            Inj Volume : Manually
Acq. Method     : C:\CHEM32\1\METHODS\XPT_120TO180_SPLIT_20.M
Last changed    : 1/19/2016 11:10:29 AM by Xiaoping
Analysis Method : C:\CHEM32\1\DATA\XPT\XPT092.D\DA.M (XPT_120TO180_SPLIT_20.M)
Last changed    : 1/19/2016 12:08:33 PM by Xiaoping
                  (modified after loading)
Method Info     : Oven: 5 min @120; 2/min to 180; 5 min @ 180
                  Inlet temp 220
                  Split ratio 20
                  Detector temp 300
```

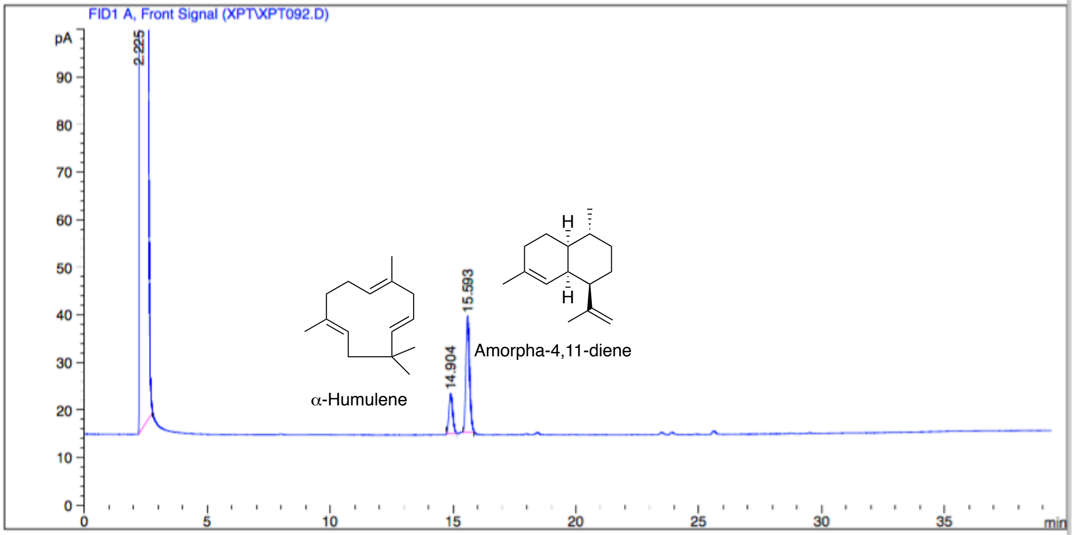


```
=====================================================================
                         Area Percent Report
=====================================================================

Sorted By             :      Signal
Multiplier            :      1.0000
Dilution              :      1.0000
Use Multiplier & Dilution Factor with ISTDs


Signal 1: FID1 A, Front Signal
```

| Peak # | RetTime [min] | Type | Width [min] | Area [pA*s] | Height [pA] | Area % |
|---|---|---|---|---|---|---|
| 1 | 2.225 | BB S | 0.0770 | 4.56939e6 | 7.20100e5 | 99.99253 |
| 2 | 14.904 | BB | 0.1584 | 88.21428 | 8.41147 | 0.00193 |
| 3 | 15.593 | BB | 0.1479 | 253.34280 | 24.45502 | 0.00554 |

```
Instrument 1 1/19/2016 12:08:35 PM Xiaoping                          Page    1 of 2

Data File C:\CHEM32\1\DATA\XPT\XPT092.D
Sample Name: XPT092

    Totals :                   4.56973e6   7.20132e5


=====================================================================
                          *** End of Report ***
```

*Table S3, entry 11:*

```
=====================================================================
Acq. Operator   : XPT
Acq. Instrument : Instrument 1                    Location :   -
Injection Date  : 4/7/2016 12:23:50 PM
                                               Inj Volume : Manually
Acq. Method     : C:\CHEM32\1\METHODS\XPT_120TO180_SPLIT_20.M
Last changed    : 4/7/2016 12:18:03 PM by XPT
Analysis Method : C:\CHEM32\1\DATA\XPT\XPT109-2.D\DA.M (XPT_120TO180_SPLIT_20.M)
Last changed    : 9/29/2016 6:00:52 PM by VG
                  (modified after loading)
Method Info     : Oven: 5 min @120; 2/min to 180; 5 min @ 180
                  Inlet temp 220
                  Split ratio 20
                  Detector temp 300
```

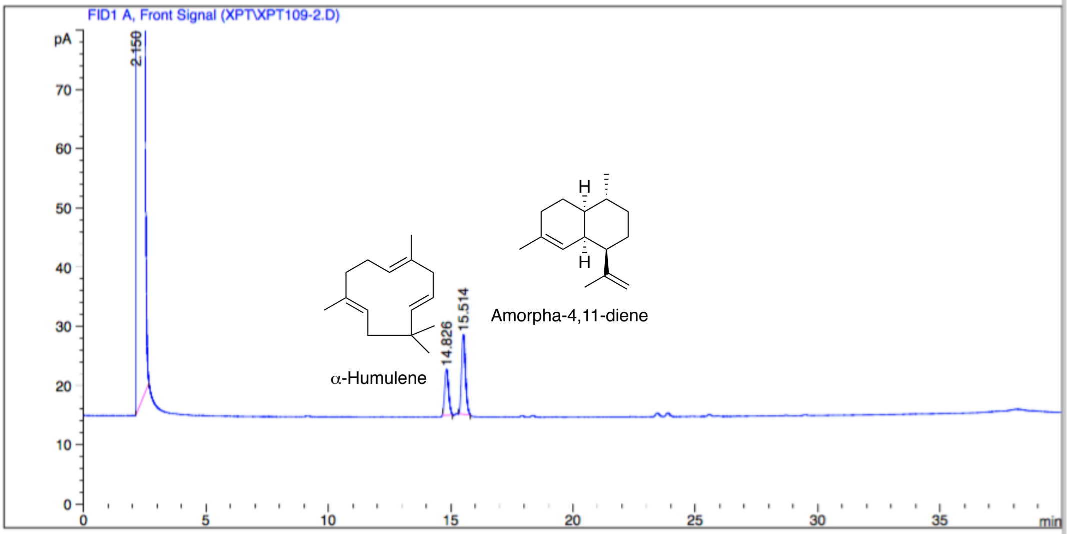


```
=====================================================================
                          Area Percent Report
=====================================================================

Sorted By             :      Signal
Multiplier            :      1.0000
Dilution              :      1.0000
Use Multiplier & Dilution Factor with ISTDs


Signal 1: FID1 A, Front Signal

Peak RetTime Type  Width     Area      Height     Area
  #   [min]        [min]   [pA*s]      [pA]         %
----|-------|----|-------|----------|----------|--------|
   1   2.150 BB S  0.0737 4.50283e6  7.43070e5  99.99479
   2  14.826 BB    0.1648   86.00275    7.78713   0.00191
   3  15.514 BB    0.1608  148.70685   13.47437   0.00330
```





```
    Totals :                  4.50306e6  7.43091e5


=====================================================================
                          *** End of Report ***
```

**References:**


[1] A. B. Woodside, Z. Huang, C. D. Poulter, *Org. Synth.* **1988**, *66*, 211.

[2] V. J. Davisson, A. B. Woodside, T. R. Neal, K. E. Stremler, M. Muehlbacher, C. D. Poulter, *J. Org. Chem.* **1986**, *51*, 4768-4779.

[3] M. J. Calvert, P. R. Ashton, R. K. Allemann, *J. Am. Chem. Soc.* **2002**, *124*, 11636-11641.

[4] M. Bradford, *Anal. Biochem.* **1976**, *72*, 248-254.
